# Enhanced Partial Discharge Signal Denoising Using Dispersion Entropy Optimized Variational Mode Decomposition

**DOI:** 10.3390/e23121567

**Published:** 2021-11-25

**Authors:** Ragavesh Dhandapani, Imene Mitiche, Scott McMeekin, Venkateswara Sarma Mallela, Gordon Morison

**Affiliations:** 1 Department of Electrical and Communication Engineering, College of Engineering, National University of Science & Technology, Seeb P.O. Box 2322, Oman; ragavesh@nu.edu.om (R.D.); vsmallela@gmail.com (V.S.M.); 2 Department of Computing, School of Computing, Engineering and Built Environment, Glasgow Caledonian University, Glasgow G4 0BA, UK; imene.mitiche@gcu.ac.uk; 3 School of Computing, Engineering and Built Environment, Glasgow Caledonian University, Glasgow G4 0BA, UK; scott.mcmeekin@gcu.ac.uk

**Keywords:** dispersion entropy, group-sparse total variation, mutual information entropy, partial discharge denoising, variational mode decomposition

## Abstract

This paper presents a new approach for denoising Partial Discharge (PD) signals using a hybrid algorithm combining the adaptive decomposition technique with Entropy measures and Group-Sparse Total Variation (GSTV). Initially, the Empirical Mode Decomposition (EMD) technique is applied to decompose a noisy sensor data into the Intrinsic Mode Functions (IMFs), Mutual Information (MI) analysis between IMFs is carried out to set the mode length *K*. Then, the Variational Mode Decomposition (VMD) technique decomposes a noisy sensor data into *K* number of Band Limited IMFs (BLIMFs). The BLIMFs are separated as noise, noise-dominant, and signal-dominant BLIMFs by calculating the MI between BLIMFs. Eventually, the noise BLIMFs are discarded from further processing, noise-dominant BLIMFs are denoised using GSTV, and the signal BLIMFs are added to reconstruct the output signal. The regularization parameter λ for GSTV is automatically selected based on the values of Dispersion Entropy of the noise-dominant BLIMFs. The effectiveness of the proposed denoising method is evaluated in terms of performance metrics such as Signal-to-Noise Ratio, Root Mean Square Error, and Correlation Coefficient, which are are compared to EMD variants, and the results demonstrated that the proposed approach is able to effectively denoise the synthetic Blocks, Bumps, Doppler, Heavy Sine, PD pulses and real PD signals.

## 1. Introduction

High Voltage (HV) equipment uses a variety of insulating materials for protecting the system and have dielectric media that are either solid, liquid, or gas depending on the design requirements of HV equipment. Insulation deterioration could advance further to physical and chemical degradation of the local and adjacent insulation, which may result in failure of the entire insulation, leading to failure of the power system equipment [1]. Due to the high voltage stress, localized dielectric breakdown of a small portion of an insulator occurs, resulting in a Partial Discharge (PD) signal. As a part of the condition-based maintenance plan for HV equipment, PD monitoring is an effective and non-destructive diagnostic tool that helps to assess the condition of HV equipment. Hence, PD measurements in generators, HV cables, motors, switch gears, transformers, etc., are carried out during operating (on-line measurement) conditions. The most common issue faced during on-line PD measurements is the interference of external signal referred as noise, which sometimes has a very high amplitude compared to the PD signal [2,3]. The most common on-site noise signals reported during PD measurements are noise from corona discharge, white Gaussian noise, thermal noise, pink noise, and high-frequency signal interference from communication equipment, commonly referred to as Discrete Spectral Interference (DSI) [4,5].

In the past few years, many researchers proposed signal decomposition based denoising algorithms in various engineering disciplines, the usage of such techniques being applied to PD signals, such as Wavelet Transform (WT) [4,5,6,7,8,9], Empirical Mode Decomposition (EMD) and its variants [10,11], Adaptive Local Iterative Filtering (ALIF) [12] and Variational Mode Decomposition (VMD) [13,14,15]. WT is the one of the most widely used decomposition method in various domains. In [4,5] the authors applied hard and soft thresholding on the coefficients of the wavelet transform, this was then extended by Ghorat et al. in [9] as Adaptive Dual Tree Complex Wavelet Transform (ADTCWT) with an automatic threshold by applying adaptive singular value decomposition. Wavelet denoising methods rely on the type and order of the wavelet, the level of decomposition, and threshold type, which needs parameter tuning and the performance depends on effective selection of those parameters. The selection of the mother wavelet is critical in the wavelet denoising method as seen in literature [5,7], the Energy Based Wavelet Selection (EBWS) method [6], Correlation Based Wavelet Selection (CBWS) method [6,8], and Signal-to-Noise Ratio (SNR) Based Wavelet Selection (SNRBWS) method [8]. Daubechies as the mother wavelet is effective in denoising PD signals mixed with DSI and white noise. However, denoising by thresholding of wavelet coefficients often introduces some artifacts such as spurious noise spikes and pseudo-Gibbs oscillation, which hinders the performance of these methods.

Apart from the usage of WT, many adaptive signal decomposition techniques were used in denoising applications [16]. Empirical Mode Decomposition (EMD) proposed by Huang et al. [17] is used in PD denoising [10], and its variant Novel Adaptive Ensemble EMD (NAEEMD) is applied for denoising PD signals [11]. The EMD method proved to be powerful in extracting IMFs from the given signal, however, other issues such as high computation time, error accumulation, mode mixing, and end effects are reported in [18]. Ensemble EMD (EEMD) [19] and Complete Ensemble EMD with Adaptive Noise (CEEMDAN) [20] has been developed based on EMD, however, all the issues mentioned above have not been fully addressed. To avoid such issues, Dragomiretskiy and Zosso [21] proposed VMD, an algorithm, which is a non-recursive decomposition method that decomposes a multi-component input signal into a set of Band Limited IMFs (BLIMFs). The PD signal is acquired through a sensor circuit that contains PD pulses with different frequency levels and various noise sources. The VMD method can decompose the sensor signal into a set of BLIMFs, which is beneficial in terms of analysing the decomposed PD signal. A recent and comprehensive review of EMD, EEMD, CEEMDAN, and VMD methods presented in [22] lists several advantages of VMD methods and suggests the VMD method for applications to the vibration-based condition monitoring of mechanical systems. The VMD has also been utilised in PD signal denoising in [13,14,15] where an optimised VMD and wavelet (OVMDW) is applied in denoising UHF PD signals [13], and the hybrid of VMD and Wavelet Packet Transform (WPT) were applied to synthetic, real-time PD signal denoising [14]. However, in both methods only white noise was addressed. The PD fault diagnosis procedure proposed in [15] has VMD as the decomposition technique followed by feature extraction from the selected IMFs to train a classifier.

The entropy proposed by Shannon [23] is an effective and widely used measure to study the randomness or uncertainty of time series data. Many forms of entropy measures are used in denoising methods. In adaptive denoising methods, Mutual Information (MI) entropy is commonly used for analyzing the frequency between IMFs to select the specific IMFs for further processing [24,25,26]. The MI of phase spectra between consecutive IMFs is presented to decide stochastic or deterministic components present in IMFs [27]. The spectral density initial IMFs of EMD are spread moderately across the frequency spectrum. However, to the best of the author’s knowledge, no experimental studies have been carried out to compare it against VMD.

Permutation Entropy (PE) is a measure for arbitrary time series based on analysis of permutation patterns [28], which reflects the complexity of the signal used in PD denoising [29], however it only considers the order of amplitude values and does not consider the differences between amplitude values. Dispersion Entropy (DE) is an improved version of PE [30], which is used for analyzing various signal properties such as amplitude, frequency and noise-power [29]. The DE along with MIE is used to identify noisy, noise-dominant, and signal-dominant IMFs for further processing.

Recently, sparse representation has become widely used in signal processing applications. Rudin et al. proposed a total variation denoising method based on an optimization problem for the removal of noise in 2-dimensional data, i.e., image [31] and T. Figueiredo et al. introduced Majorization-Minimization (MM) algorithm for denoising an image [32]. Selesnick and Chen and Condat [33,34] proposed an one dimensional Total Variation Denoising (TVD) algorithm applied in vibration signal denoising [35] and partial discharge signal denoising [3,36]. Selesnick and Chen proposed an iterative Group-Sparse Total Variation (GSTV) algorithm derived using MM optimization method, which is suitable when the estimated signal to the group is sparse. GSTV is designed to alleviate the staircase artifact often arising in the TVD. GSTV is computationally efficient due to the use of fast solvers for the banded system, and applied for PD signal denoising in [37].

In this paper, a new denoising method, VMD-GSTV, is presented to remove the various contaminating noise sources from partial discharge signals. A comparative analysis of EMD-Detrended Fluctuation Analysis (EMD-DFA) [38], Complete Ensemble EMD with Adaptive Noise (CEEMDAN) [20], EMD-GSTV, and VMD-GSTV are conducted, and the algorithms are applied to the simulated synthetic signals and real PD data. The performance indices of these algorithms are computed and presented.

The rest of this paper is organised as follows: PD measurement setup and source of disturbances in PD measurement are discussed in Section 2. The proposed method followed by the techniques used such as VMD, MI, DE, and GSTV methods are briefly introduced in Section 3. Applications of the VMD-GSTV to synthetic and real-world signals are presented in Section 4. The simulation results and testing are presented in Section 5, discussion and conclusion are in Section 6 and Section 7, respectively.

## 2. PD Measurement

**Figure 1:**
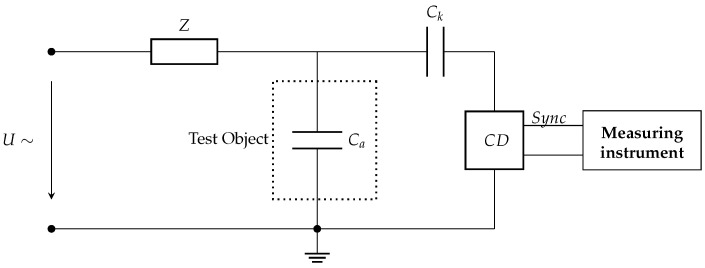
Standard setup used for PD measurement [39].

**Figure 2:**
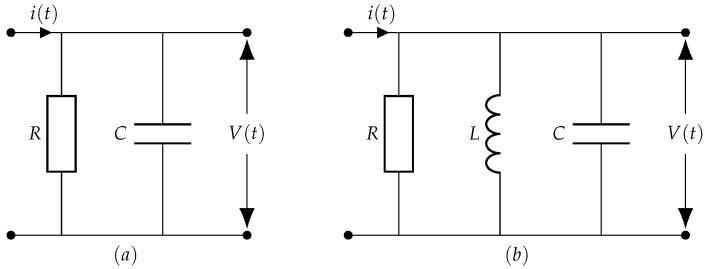
Impedance matching device. (**a**) RC Coupling device. (**b**) RLC Coupling device.

According to the IEC60270 standard [39], the most common techniques used for PD measurement are electrical, ultra-high frequency measurement, acoustic emission, and the high frequency current transformer (HFCT) sensor method. Each type exhibits a different behavior in terms of pulse type, width, rise, and decay time. A typical electric PD measurement setup with the test object is shown in Figure 1, which is one of the most popular methods used in controlled areas such as laboratories. In the PD measurement setup, *U*∼ is the high-voltage power supply, *Z* is the filter, and $C_{a}$ is the test object. The coupling capacitor $\left(C_{k}\right)$ allows flow of short PD current pulse, and the matching impedance $\left(Z_{m}\right)$ of Coupling Device (CD) converts the PD current pulses into voltage pulses. The matching impedance, $Z_{m}$ is either RC circuit for wide-band PD detection or RLC circuit for narrow-band detection as shown in Figure 2. The detector outputs different pulse shapes based on the type of detection circuit, which is realized as the natural response of either parallel RC or RLC circuit [7,40]. These are Damped Exponential Pulse (DEP) and Damped Oscillatory Pulse (DOP).

### Sources of Disturbances in PD Measurement

A typical PD measurement system contains a sensor, an amplifier, an oscilloscope, and a computer for data acquisition and processing of data. HFCTs are often used as sensor in non-invasive PD measurement systems, which is clamped around the conductor that connects the cable to the ground. According to the IEC60270 standard [39], the main source of noise in PD measurement is background noise, which does not originate in the test object. Background noise comes in the form of either white noise in the measurement system, high-frequency signal interference from radio broadcasts, or due to the switching operations in other circuits or commutating machines, etc. [39]. The external interference in PD measurements can lead to the wrong detection of PD signals due to the larger magnitude than the PD signal. The on-site noise and disturbance can be classified as:

- White noise—a random noise signal that has same power at all frequencies. The thermal noise generated by the detection system and the noise sources such as ambient noise and amplifier noise are considered as white noise. a detailed discussion can be found in [2,5,41];
- DSI—the interference from radio transmission such as communication and amplitude modulation/frequency modulation ratio emissions. A detailed discussion is found in [7];
- Pink (or $\frac{1}{f}$) noise is a signal with a power spectral density that is inversely proportional to the frequency of the signal. Detailed discussion can be found in [24].

In this work, DOP and DEP models along with white noise, DSI, and color noise is considered for implementation. Apart from this, the proposed denoising method is evaluated using the standard test signals such as Blocks, Bumps, Doppler, Heavy Sine, and real PD data. The simulation models with the parameter settings will be discussed later in this paper.

## 3. Review of Algorithms

**Figure 3:**
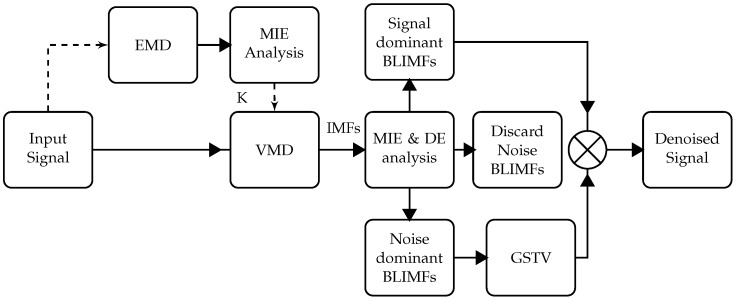
Overall process flow diagram of the proposed VMD-GSTV denoising method.

Figure 3 outlines the method followed in the proposed denoising method VMD-GSTV. The proposed VMD-GSTV inherits the advantages of both the VMD and GSTV method in addition to mutual information and dispersion entropy. The steps of the algorithm are as follows:

1. Decompose the input signal by the EMD to obtain IMFs;
2. Calculate the MI of the phase spectra of the IMFs and determine the number of modes (*K*) for VMD;
3. Decompose the input signal by VMD using the mode parameter *K* to obtain BLIMFs;
4. Calculate the MI of the phase spectra of the BLIMFs, and draw the boundary between noise and noise-dominant BLIMFs;
5. Compute the DE of noise-dominant BLIMFs and set the value of λ for GSTV;
6. Denoise the noise-dominant BLIMFs with GSTV to create an output vector and discard the noise BLIMFs;
7. Add the signal BLIMFs to the output vector directly without denoising to reconstruct the signal.

In the following sections, the techniques that formulate the proposed denoising method are reviewed and discussed.

### 3.1. Variational Mode Decomposition (VMD)

VMD decomposes a real valued input signal f into *K* number of predefined BLIMFs referred to as $u_{k}$, which is compact around a central frequency $\omega_{k}$. The process of signal decomposition to solve a constrained variational problem is written as [21]:

(1) $$\min_{\left\{u_{k}\right\},\left\{\omega_{k}\right\}}\left\{\sum_{k=1}^{K}{∥\frac{\partial }{\partial_{t}}\left[\left(\delta \left(t\right)+\frac{j}{\pi t}\right)\times u_{k}\left(t\right)\right]e^{-j\omega_{k}t}∥}_{2}^{2}\right\}\;\mathrm{subject}\;\mathrm{to}\;f\left(t\right)=\sum_{k=1}^{K}u_{k}\left(t\right)$$

where $\left\{u_{k}\right\}=\left\{u_{1},\cdots ,u_{K}\right\}$ and $\left\{\omega_{k}\right\}=\left\{\omega_{1},\cdots ,\omega_{K}\right\}$ are the mode components and their center frequencies, respectively, *K* is the total number of modes to be recovered, δ(t) denotes the impulse function and f is the input signal. To solve Equation (1), constrained variational problem is transformed into unconstrained. This is achieved by introducing the Lagrangian multiplier (λ) and quadratic penalty term α. The new unconstrained problem is as follows:

(2) $$\begin{array}{c} \;L\left(\left\{u_{k}\right\},\left\{\omega_{k}\right\},\lambda \right)=\;\alpha \sum_{k=1}^{K}{∥\frac{\partial }{\partial_{t}}\left[\left(\delta \left(t\right)+\frac{j}{\pi t}\right)\times u_{k}\left(t\right)\right]e^{-j\omega_{k}t}∥}_{2}^{2}\\ \;+{∥f\left(t\right)-\sum_{k=1}^{K}u_{k}\left(t\right)∥}_{2}^{2}+\langle \lambda \left(t\right),f\left(t\right)-\sum_{k=1}^{K}u_{k}\left(t\right)\rangle \end{array}$$

The solution of Equation (2) can now be seen as the saddle point of the augmented Lagrangian in a sequence of iterative sub-optimizations referred to as the Alternate Direction Method of Multipliers (ADMM) [42]. The optimization procedure of VMD includes the following steps:

1. Initialize modes $\left\{{\hat{u}}_{k}^{1}\right\}$, center frequency $\left\{\omega_{k}^{1}\right\}$, and ${\hat{\lambda }}^{1}$. Set n=0
2. Update the modes ${\hat{u}}_{k}$ for all ω≥0: ${\hat{u}}_{k}^{n+1}\left(\omega \right)=\frac{\hat{f}\left(\omega \right)-\sum_{i<k}{\hat{u}}_{i}^{n+1}\left(\omega \right)-\sum_{i>k}{\hat{u}}_{i}^{n}\left(\omega \right)+\frac{{\hat{\lambda }}^{n}\left(\omega \right)}{2}}{1+2\alpha {\left(\omega -\omega_{k}^{n}\right)}^{2}}$
3. Update the center frequencies $\omega_{k}$: $\omega_{k}^{n+1}=\frac{\int_{0}^{\infty }\omega {\left|{\hat{u}}_{k}^{n+1}\left(\omega \right)\right|}^{2}d\omega }{\int_{0}^{\infty }{\left|{\hat{u}}_{k}^{n+1}\left(\omega \right)\right|}^{2}d\omega }$
4. Update dual ascent for all ω≥0:${\hat{\lambda }}^{n+1}\left(\omega \right)\leftarrow {\hat{\lambda }}^{n}\left(\omega \right)+\tau \left(\hat{f}\left(\omega \right)-\sum_{k}{\hat{u}}_{k}^{n+1}\left(\omega \right)\right)$
5. Repeat steps 2–4, until convergence: $\frac{\sum_{k}{∥{\hat{u}}_{k}^{n+1}-{\hat{u}}_{k}^{n}∥}_{2}^{2}}{{∥{\hat{u}}_{k}^{n}∥}_{2}^{2}}<\epsilon$

During the optimization procedure, the VMD method follows a non-recursive approach to obtain the BLIMFs and the quadratic data fidelity term in Equation (2) improves the convergence rapidly. Further details and mathematical description of the VMD algorithm can be found in [21]. Within this paper the input signal is decomposed into *K* predefined modes with the parameters, α = 2000 and τ = 0, tolerance level set as $1\times 10^{-6}$ as described in [21]. The improper selection of the number of modes *K* results in over- or under-decomposition. In this work we avoid this potential issue by selecting *K* using an EMD-based algorithm using mutual information analysis.

**Figure 4:**
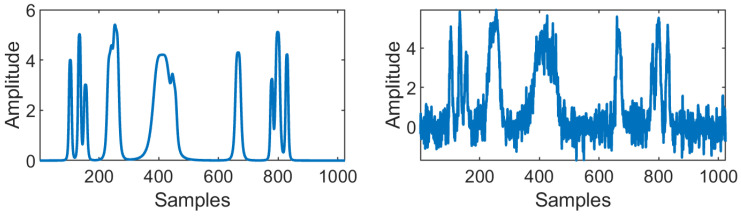
The image on the (**left**) shows synthetic ’Bumps’ signal of 1024 samples and the image on (**right**) shows noisy ‘Bumps’ signal with added white Gaussian noise of 10 dB.

**Figure 5:**
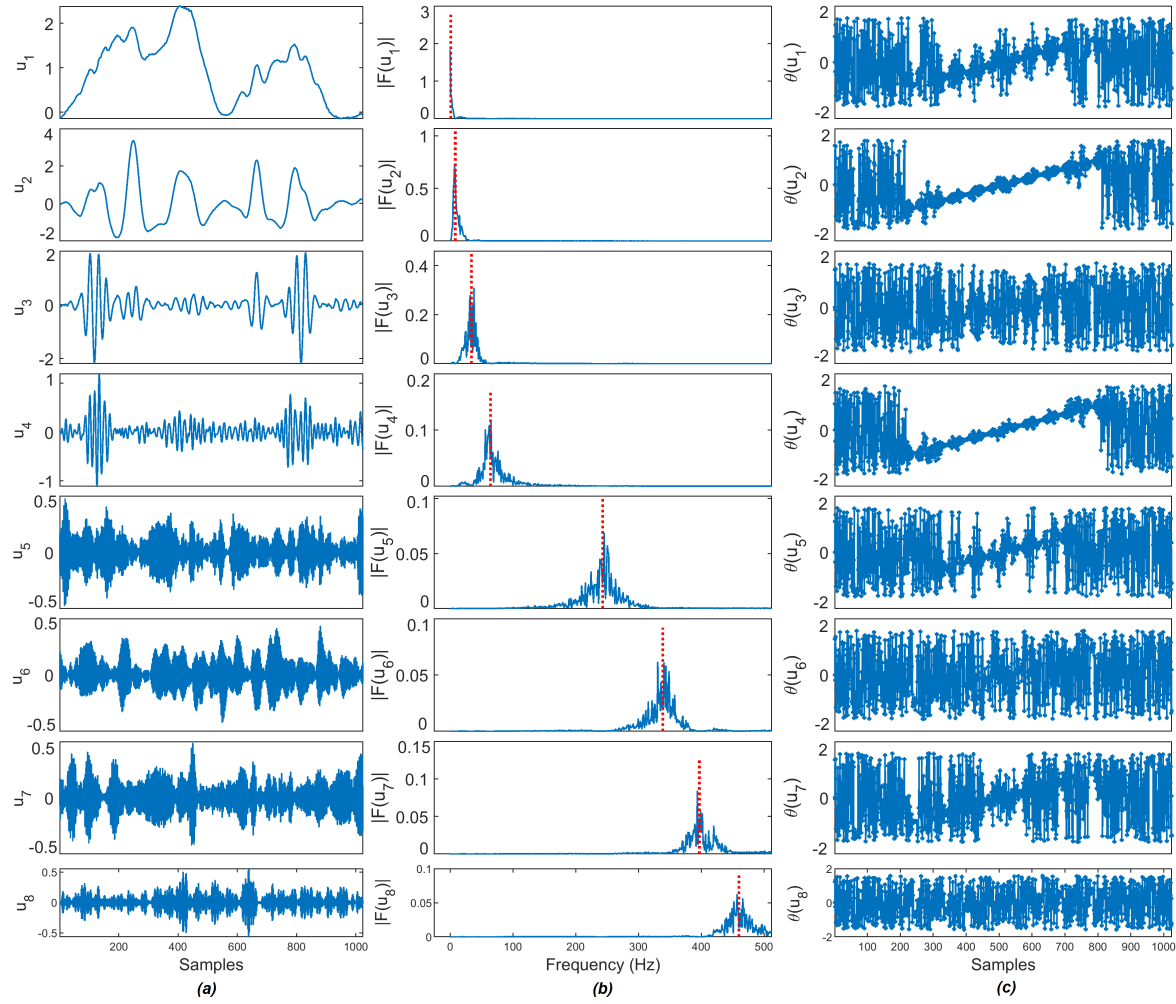
The signal decomposition results of the Figure 4: (**a**) BLIMFs extracted using VMD; (**b**) frequency spectra and (**c**) phase spectra.

**Figure 6:**
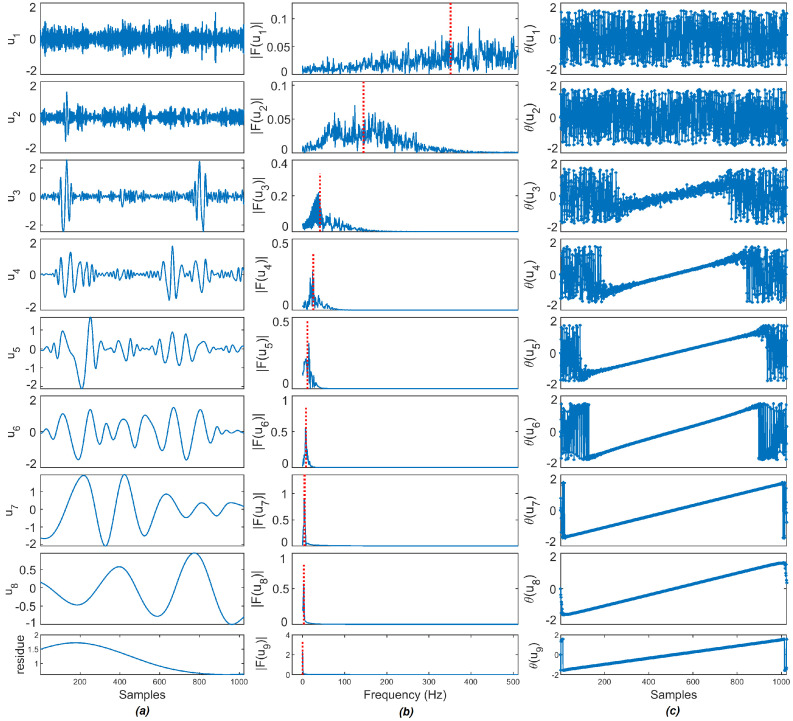
The signal decomposition results of the Figure 4: (**a**) IMFs extracted using EMD; (**b**) frequency spectra and (**c**) phase spectra.

To demonstrate the VMD decomposition for a noisy input signal f is decomposed using VMD resulting into a set of BLIMFs ($u_{k})$, then the frequency spectra (|$F(u_{k})$|) and the phase spectra ($\theta (u_{k})$) of BLIMFs are calculated. As an example, a sample synthetic ‘Bumps’ signal with added white Gaussian noise of 10 dB is shown in Figure 4 and the noisy ‘Bumps’ signal is decomposed using VMD as shown in Figure 5, in line with the literature [27], the noisy ‘Bumps’ signal is also decomposed using EMD as shown in Figure 6.

### 3.2. Mutual Information (MI)

Shannon [23] developed MI to measure the dependency between two random variables. For example, if two random variables are strictly said to be independent, their MI is zero. Let x and y be two independent random variables on same sample space X and Y, respectively, then MI can be defined as:

(3) $$I\left(x,y\right)=\sum_{y\in Y}\sum_{x\in X}p\left(x,y\right)\log\left(\frac{p(x,y)}{p(x)p(y)}\right)$$

where p(x,y) is the joint Probability Density Function (PDF) of x and y, p(x) and p(y) are the marginal PDF of x and y, respectively. Moreover, the MI can be expressed as

(4) I(x,y)=H(x)+H(y)−H(x,y)

where H(x) and H(y) are information entropy and H(x,y) is joint entropy of x and y.

According to Rios and De Mello [27], the stochastic or deterministic components present in IMFs can be decided by finding the MI of phase spectra between consecutive IMFs. A noisy signal decomposed by EMD has a set of IMFs in order from high-frequency to low-frequency IMFs. Two consecutive high-frequency IMFs exhibit a lower level of mutual information, as they are considered to be stochastic. Two consecutive low-frequency IMFs shares high information between them resulting in higher values of MI, hence it is considered to be deterministic. As observed in Figure 6, the frequency is reduced as new IMFs are extracted in the EMD method. In Figure 5, the frequency of BLIMFs is increased as new IMFs are extracted in the VMD method. The mean frequency and MI of phase spectra of the IMFs and BLIMFs are listed in Table 1 and Table 2, respectively.

The threshold value selected for EMD is 0.9 and the VMD is 0.6 for dividing noise and noise-dominant IMFs. These values are selected based on the mean MI values between IMFs of various noise sources discussed in Section 4.4. As shown in Table 2, the first value above the threshold is used to draw the boundary. The MI value 2.3165 of $IMF_{4-5}$ is above the threshold value 0.9 for EMD and 1.4764 of $IMF_{1-2}$ is above the threshold value 0.6 for VMD, therefore $IMF_{5}$ and $IMF_{2}$ are selected as the boundary between noise and signal-dominant IMFs in EMD and VMD methods, respectively. Then, each IMF is analyzed using the DE as summarized in the following section.

### 3.3. Dispersion Entropy (DE)

In this work, the DE measure is used to classify whether a particular IMF is a noise or noise-dominant or signal IMF. PE is a measure for arbitrary time series based on analysis of permutation patterns and DE is an improved version of PE to quantify the regularity of time series [30]. For a given time series x with the length of *N*, the DE is calculated as follows [30]: Initially, $x=x_{1},x_{2},\cdots ,x_{N}$ are mapped to $y=y_{1},x_{2},\cdots ,y_{N}$ from 0 to 1 using the Normal Cumulative Distribution Function (NCDF) which is defined as:

(5) $$y=\frac{1}{\sigma \sqrt{2\pi }}\int_{-\infty }^{x}e^{\frac{-{\left(t-\mu \right)}^{2}}{2\pi \sigma^{2}}}dt$$

where (μ) is the mean and (σ) is the standard deviation of the signal x, and y is the probability that a random variable x is less than or equal to the time series x. Then each $y_{j}$, where j=1,2,⋯,N are assigned a class from 1 to *c* by the linear algorithms as follows:

(6) $$z_{j}^{c}=\mathrm{round}\left(c\cdot y_{j}+0.5\right)\;j=1,2,\ldots ,N$$

where $z_{j}^{c}$ denotes the *j*-th member of the classified time series.

Then, the embedding vector $z_{i}^{m,c}$ with dimension *m* and time delay *d* are generated using the following equation:

(7) $$z_{i}^{m,c}=\left\{z_{i}^{c},z_{i+d}^{c},\ldots ,z_{i+(m-1)d}^{c}\right\}\;i=1,2,\ldots ,N-\left(m-1\right)d$$

then, each time series $z_{i}^{m,c}$ is mapped to a dispersion pattern $\pi_{v_{0}v_{1}\cdots v_{m-1}}$, where $v_{0}=z_{i}^{c}$, $v_{1}=z_{i+d}^{c}$, *…*, $v_{m-1}=z_{i+(m-1)d}^{c}$. The total possible number of dispersion patterns is $c^{m}$.

The relative frequency of each potential dispersion patterns can be given by:

(8) $$p\left(\pi_{v_{0}v_{1}\cdots v_{m-1}}\right)=\frac{\;\#\left\{i|i\leq N-\left(m-1\right)d,z_{i}^{m,c}\;\mathrm{has}\;\mathrm{type}\;\pi_{v_{0}v_{1}\ldots v_{m-1}}\right\}}{N-(m-1)d}$$

where # means the number of dispersion patterns of $\pi_{v_{0}v_{1}\cdots v_{m-1}}$ that is assigned to $z_{i}^{m,c}$.

Lastly, according to Shannon’s definition of entropy, the DE value with embedding dimension *m*, the number of classes *c*, and time delay *d* is computed as follows:

(9) $$\mathrm{DE}\left(x,m,c,d\right)=-\sum_{\pi =1}^{c^{m}}p\left(\pi_{v_{0}v_{1}\ldots v_{m-1}}\right)\cdot \ln\left(p\left(\pi_{v_{0}v_{1}\ldots v_{m-1}}\right)\right)$$

**Figure 7:**
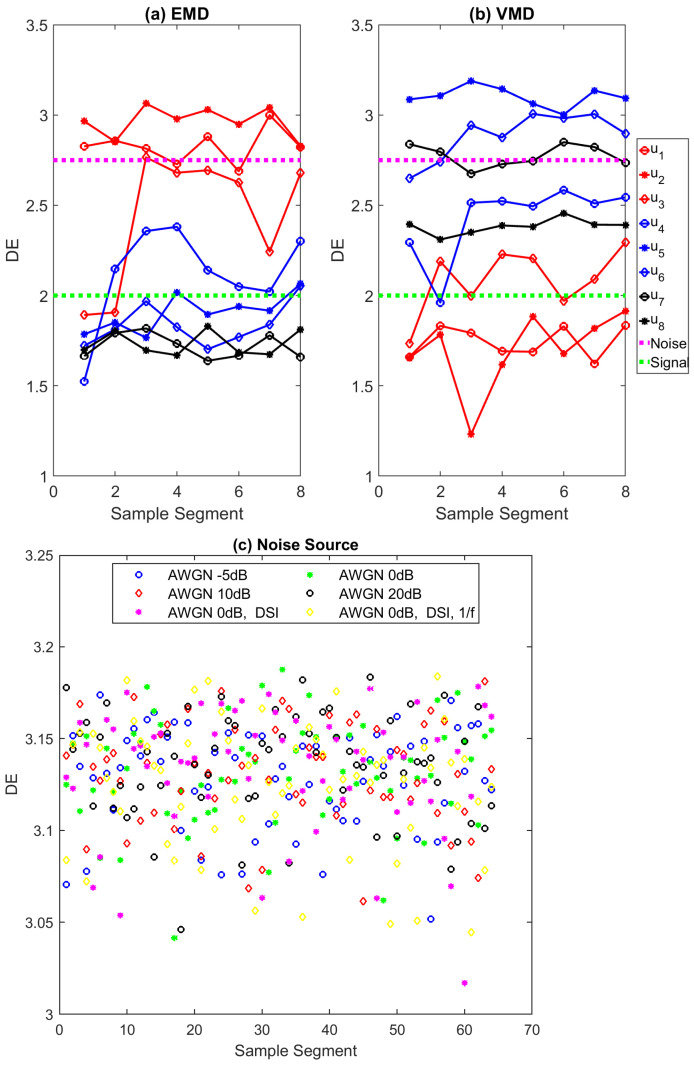
Dispersion entropy of (**a**,**b**) EMD and VMD IMFs obtained for the ‘Bumps’ signal shown in Figure 4 and (**c**) three noise sources such as AWGN, DSI, and 1/f noise. In (**a**,**b**), the segments above the ‘Noise’ line are considered as noise IMFs, the segments below the ‘Signal’ line are considered as signal IMFs and the segments in between are referred to as noise-dominant IMFs.

As suggested in [30], the following parameters are selected: the embedding dimension *m* = 2, the number of classes *c* = 5, and the time delay *d* = 1. Each IMF obtained through EMD and VMD methods are segmented into 128 sample frames and the DE value of each segment is calculated. The segment size is selected as the power of 2 value and $c^{m}<N$. For example, the ‘Bumps’ signal of the 1024 sample point that is decomposed through EMD and VMD has eight IMFs, each IMF is further segmented into eight frames, and the DE of each frame is calculated. The computed DE value of each frame of EMD and VMD is shown in Figure 7a,b. Three noise sources, as described in Section 4.4, such as Additive White Gaussian Noise (AWGN), DSI, and the color noise of 8K sample points are segmented into 128 sample frames, then the DE value of each sample frame is calculated and plotted in Figure 7c. It is observed that the DE values of various noise sources are higher than three for all segments. As observed in Figure 7a,b, the DE values of noise IMFs’ segments are higher than 2.75, and in some cases above 3 as well. On the other hand, the DE values of signal IMFs’ segments are in the range of 1 to 2 and the DE values of noise-dominant IMFs are in the range of 2 to 2.75. The noise-dominant IMFs need to be filtered based on the noise content present in the IMFs, and hence based on these DE values of each IMF, the key parameter is set for the denoising method.

### 3.4. Group-Sparse Total Variation (GSTV) Denoising

GSTV is an extension of total variation denoising, designed to reduce the staircase artifact that occurs in the total variation denoising method. A suitable penalty function is used to promote the group sparsity behavior of the signal derivative. A computationally efficient and fast converging MM algorithm is used to minimize the *F(x)* without any parameters [33].

Assume that noisy signal $y_{n}\in R^{N}$ is modeled as given in (10)

(10) $$y_{n}=x_{n}+w_{n}\;n=1,2\ldots ,N$$

$x_{n}\in R^{N}$ and $w_{n}\in R^{N}$ are the clean signal and the noise, respectively. The clean signal can be estimated by solving the optimization problem:

(11) $$x^{*}=\arg\min_{x\in R^{N}}\left\{F\left(x\right)=\frac{1}{2}{∥y_{n}-x_{n}∥}_{2}^{2}+\lambda \phi \left(Dx_{n}\right)\right\}$$

where ϕ is a penalty function that promotes group sparsity, λ is the regularization parameter and ${\mathrm{Dx}}_{n}$ as the first-order difference matrix of an N-point signal $x_{n}$, where D is a matrix of size (N−1)×N is represented as

(12) $$\;D=\left[\begin{array}{cccccc} -1 & 1 & & & \\ & -1 & 1 & & \\ & & \ddots & \ddots & \\ & & & -1 & 1\\ & & & & -1 & 1 \end{array}\right]$$

The penalty function described in [33] is used in this work,

(13) $$\phi \left(v\right)=\sum_{n}{\left[\sum_{p=0}^{P-1}{\left|v\left(n+p\right)\right|}^{2}\right]}^{1/2}$$

*P* denotes the group size. The value of the *P* in the range of 1 to 10. If P=1, $\phi \left(v\right)={∥v∥}_{1}$ then Equation (11) is the standard 1D total variation denoising problem. In this work, *P* is set as 3, the function ϕ(v) is a convex measure of group sparsity. The λ has more influence on denoising as it changes the total variation of the signal. A positive value of λ is selected for denoising noise-dominant IMFs based on the DE value as described in the previous section and *P* value is selected according to the 1D denoising illustration given in [43].

## 4. Applications of the VMD-GSTV to Synthetic and Real-World Applications

**Figure 8:**
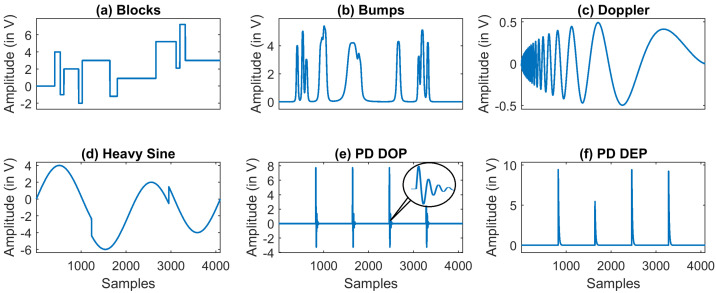
Synthetic test signals used in this work of 4 K sample points.

To validate the performance of the proposed method, we used six signal types as shown in Figure 8. As per the literature [24,26], four of the six signals are standard test signals used for evaluating denoising methods. The two other signals are DOP and DEP, which are the types of PD signals discussed in Section 2. These signals are corrupted by artificially generated noise signals. Apart from these signals, three real PD data set from [44] are used to validate the proposed method. In this section, the standard test signals, noise models, and real PD data set used in this work are presented.

### 4.1. Synthetic Test Signal Models

The test signal function in MATLAB^®^ such as Blocks, Bumps, Doppler, and Heavy Sine are used to model the synthetic signals, as shown in Figure 8a–d. The signal size is set from 1 K ($2^{10}$, 1024) to 16 K ($2^{14}$, 16,384) in order to analyze the performance of the denoising algorithms for different signal size.

### 4.2. Synthetic PD Models

Two types of partial discharge models were considered for the simulation. The PD signal $PD_{DOP}\left(t\right)$ and $PD_{DEP}\left(t\right)$ are modeled using the Equations (14) and (15).

(14) $$PD_{DOP}\left(t\right)=A\left(e^{-\alpha t}-e^{-\beta t}\right)\sin\left(\omega t\right)$$

(15) $$PD_{DEP}\left(t\right)=A\left(e^{-\alpha t}-e^{-\beta t}\right)$$

where *A* represents the amplitude of the pulse, α and β are the damping factors and ω is the damping frequency. Figure 8e,f shows the $PD_{DOP}\left(t\right)$ and $PD_{DEP}\left(t\right)$ pulse with amplitude between 3 V to 7 V, α is set as $7.5\times 10^{5}$, β is set as $16\times 10^{6}$ and ω as 150 kHz. The sampling frequency $f_{s}$ is set to 20 MHz.

### 4.3. Real PD Data

The real PD data available at [44] of the void, surface, and corona discharge signals were used to test the algorithm and the outcomes are reported.

### 4.4. Common Noise Models in Measurement System

The noise sources are independently modeled as described in the following sections and are added together to generate different noise signals as shown in Table 3. The noise signal $Sx_{n}$ is modeled with the presence and absence of AWGN, DSI, and pink noise, where *x* is 1 ⋯ 11 and *N* is the length of the signal:

(16) $$Sx_{n}=Sa_{n}+Sd_{n}+Sp_{n}$$

The six test signals are added with five levels of AWGN from −5 dB to 20 dB with the presence and absence of DSI and pink noise, making a total of 66 signals.

#### 4.4.1. Additive White Gaussian Noise (AWGN)

A more common random noise process is white noise, which possesses uniform power at all frequencies and the noise voltage amplitude has a Gaussian or Normal distribution. The MATLAB^®^ built-in function ‘*awgn*’ is used to add white Gaussian noise with a Signal-to-Noise Ratio (SNR) of −5 dB, 0 dB, 5 dB, 10 dB and 20 dB. The signal power is measured before adding the noise to it. The noise signal $Sa_{n}$ represented as S1 to S5 with five levels of AWGN.

#### 4.4.2. Discrete Spectral Interference (DSI)

The noise due to the interference of communication equipment in the form of Amplitude Modulation (AM) radio, Frequency Modulation (FM) radio, and mobile communication emissions is referred to as DSI. The frequency band of FM and mobile communications systems indicates that these systems have very minimal impact on PD measurements. The presence of continuous sinusoidal noise from the communication systems is represented in the form of a combination of AM signals given by Equation (17).

(17) $$s\left(t\right)=\sum_{c=1}^{9}A_{c}\left(1+m\times \sin\left(2\pi f_{m}t\right)\right)\times \sin\left(2\pi f_{c}t\right)$$

where $A_{c}$ is the carrier amplitude and $f_{c}$ is frequency of the carrier signal, *m* is the modulation depth, and $f_{m}$ the frequency of the modulating wave. In the simulation, the following values were used: $A_{c}=1$, $f_{c}=0.1-1.7$ MHz in steps of 200 kHz, m=0.4 and $f_{m}=1$ kHz. The s(t) is sampled with a frequency $f_{s}$ as 20 MHz to form $Sd_{n}$.

#### 4.4.3. Pink Noise

A third noise model is pink noise, simulated using MATLAB^®^ function ColoredNoise for required length, represented as $Sp_{n}$.

## 5. Numerical Experiments

The simulation is carried out using MATLAB^®^ installed on a Windows 8.1 operating system running on an Intel(R) Core i7-4700MQ CPU @ 2.40 GHz processor with 8 GB RAM. The simulated signal is added with various noise signals such as AWGN, DSI, and Color noise, denoised using various algorithms and the filter evaluation parameters were calculated for each method and compared with the proposed method.

### 5.1. Denoising Algorithms

Using simulated data, the following methods have been considered for comparing the performance of proposed method:

- EMD-DFA [38]—EMD-based denoising technique with detrended fluctuation analysis (DFA) to define the threshold to reject noisy IMFs and reconstruct the signal;
- CEEMDAN [20]—a complete ensemble EMD with adaptive noise, a variation of EEMD provides better spectral separation of the IMFs. EEMD proposed in [19] is an extension of EMD, developed to overcome the mode mixing issues;
- EMD-GSTV—classical EMD method [17] with a proposed framework for the selection of IMFs to reconstruct the signal.

### 5.2. Filter Evaluation Parameters

In order to assess the performance of denoising algorithms, the following parameters were computed and analyzed. Let us consider input signal $x_{n}$, reconstructed signal ${\mathrm{xr}}_{n}$, and noisy signal $y_{n}$.

A. Root Mean Square Error (RMSE): The RMSE between the input signal and reconstructed signal is given in Equation (18). The lower value of RMSE indicates that the reconstructed signal is similar to the simulated signal and better denoising algorithm.

(18) $$\mathrm{RMSE}=\sqrt{\frac{1}{N}\sum_{n=1}^{N}{\left(x_{n}-{\mathrm{xr}}_{n}\right)}^{2}}$$

B. Signal to Noise Ratio (SNR): The SNR is calculated in order to test the effectiveness of denoising techniques. The SNR is given as

(19) $$\mathrm{SNR}=10\times log\frac{\sum_{n=1}^{N}{\mathrm{xr}}_{n}^{2}}{\sum_{n=1}^{N}\left(y_{n}-{\mathrm{xr}}_{n}^{2}\right)}$$

The positive value of the SNR indicates the high power of the signal as compared to noise level and vice versa.

C. Correlation Coefficient (CC): It is computed using the Equation (20), where $\bar{x}$ is the mean value of $x_{n}$, $\bar{xr}$ is the mean value of ${\mathrm{xr}}_{n}$, is given by

(20) $$\mathrm{CC}=\frac{\sum_{n=1}^{N}\left(x_{n}-\bar{x}\right)\left({\mathrm{xr}}_{n}-\bar{xr}\right)}{\sqrt{\sum_{n=1}^{N}{\left(x_{n}-\bar{x}\right)}^{2}{\left({\mathrm{xr}}_{n}-\bar{xr}\right)}^{2}}}$$

The inference obtained from the CC is as follows: If CC = 1 indicates the highest shape similarity, whereas if CC = –1 means total asymmetry between the signals.

## 6. Results

The performance of denoising methods are verified using standard filter evaluation parameters such as SNR, RMSE, and CC. The results presented in Table 4, Table 5, Table 6 and Table 7 show the mean value of the parameters measured for 10 iterations of the denoised signals of various denoising algorithms. The effective filter should remove the unwanted noise components, which have no relationship to the signal of interest. The best evaluation parameters in the results tables are highlighted in bold for each method and noise levels. The analysis is carried out based on the output SNR values, signal length, RMSE, and CC. The performance parameter with high SNR, high CC, and low RMSE values are considered to be the best filters.

Noise sources such as DSI and color noises are also added with the test signals and the performance of the algorithms are evaluated. The impact of these noises imposed on the test signals are minimum and in most of the cases, the merit of the parameters remains the same.

### 6.1. Illustration of Denoising Simulated Signals Corrupted by White Noise

**Figure 9:**
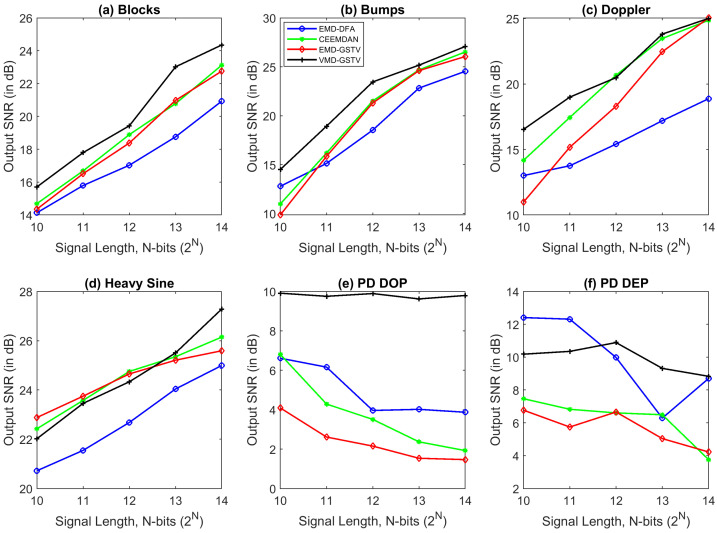
The output SNR value of various denoising algorithms at different signal length ($N=2^{10}$ to $2^{14}$) of synthetic test signals corrupted with 10 dB white noise.

**Figure 10:**
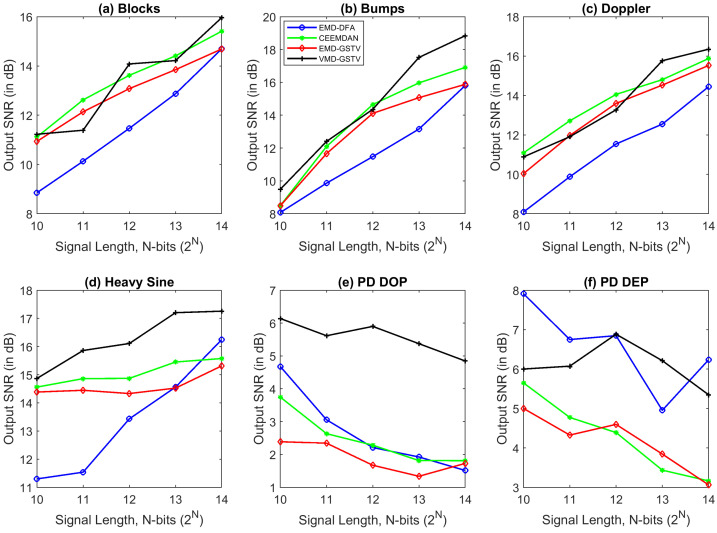
The output SNR value of various denoising algorithms at different signal length ($N=2^{10}$ to $2^{14}$) of synthetic test signals corrupted with 0 dB white noise.

**Figure 11:**
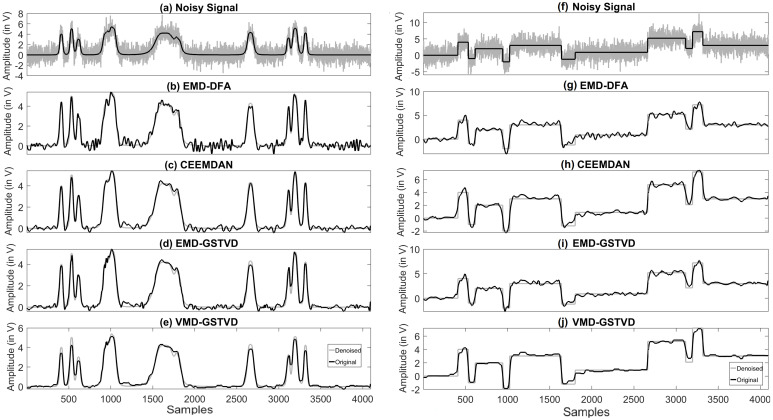
Input synthetic and noisy (**a**) ‘Bumps’ signal; (**f**) ‘Blocks’ signal, reconstructed (**b**–**e**) ‘Bumps’ signal and (**g**–**j**) ‘Blocks’ signal using various algorithms for input SNR=5 dB and signal length $N=2^{12}$.

Four out of the six signals shown in Figure 8 are the standard test signals, and the two other signals are PD signals. Table 4 presents SNR, RMSE, and CC values of denoised ‘Blocks’ signal with 16 K sample points corrupted with AWGN for various denoising methods used in this paper. Each test signal with varying signal lengths ($N=2^{10}$ to $2^{14}$) are corrupted by AWGN with input SNR −5 dB, 0 dB, 5 dB, 10 dB, and 20 dB. We can note that VMD-GSTV performs better as compared to other techniques. It is also observed from the signal length versus output SNR plots, as shown in Figure 9 and Figure 10, that VMD-GSTV provides better output SNR values for most of the synthetic test signals. A sample denoised signal ‘Bumps’ and ‘Block’ obtained from various methods are shown in Figure 11a,f.

Generally, EMD and VMD-based denoising methods exhibit lower performance in piece-wise constant signals [24,26]. Referring to Table 4, the highest output SNR value of the proposed VMD-GSTV for the ‘Blocks’ signal for all input SNR levels are followed by CEEMDAN and EMD-GSTV. The output SNR values are better in ‘Blocks’, ‘Bumps’, ‘Doppler’, and ‘PD DOP’ signals for a different signal length ($N=2^{10}$ to $2^{14}$). These result indicate the efficacy of the proposed denoising method.

Signal length plays an important role also in denoising algorithms, the computation time is directly related to the signal length. From the observation of SNR and RMSE plots, most of the algorithms perform better in higher signal length, notably VMD-GSTV is good in all synthetic test signals except PD DEP signal.

The output signals are analyzed qualitatively by plotting the original signal and denoised signals obtained from various methods. Figure 11a,f has the original ‘Bumps’ and ‘Block’ signal shown in black color and noisy ‘Bumps’ and ‘Block’ signal corrupted with input SNR=5 dB AWGN is shown in gray color. Figure 11b–e,g–j has a denoised signal of a different algorithm with the original input signal. The figures demonstrate that the proposed VMD-GSTV method has better reconstructed output signals under extreme noise conditions as compared to EMD-DFA, CEEMDAN, and EMD-GSTV methods.

**Figure 12:**
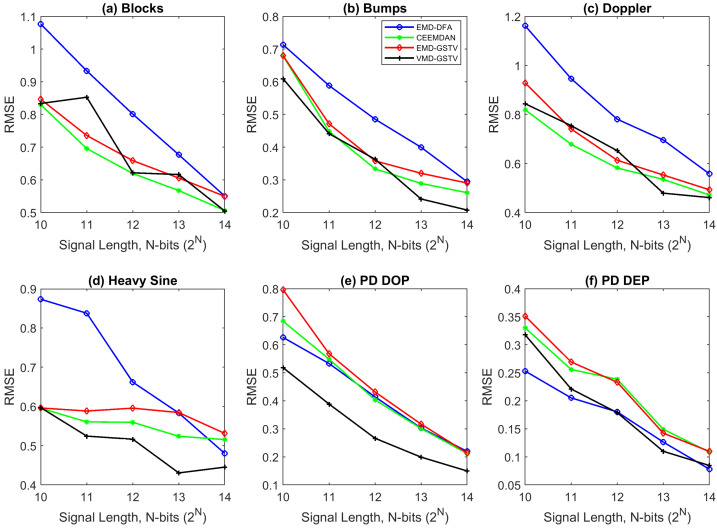
The RMSE value of various denoising algorithms at different signal length ($N=2^{10}$ to $2^{14}$) of synthetic test signals corrupted with 0 dB white noise.

**Figure 13:**
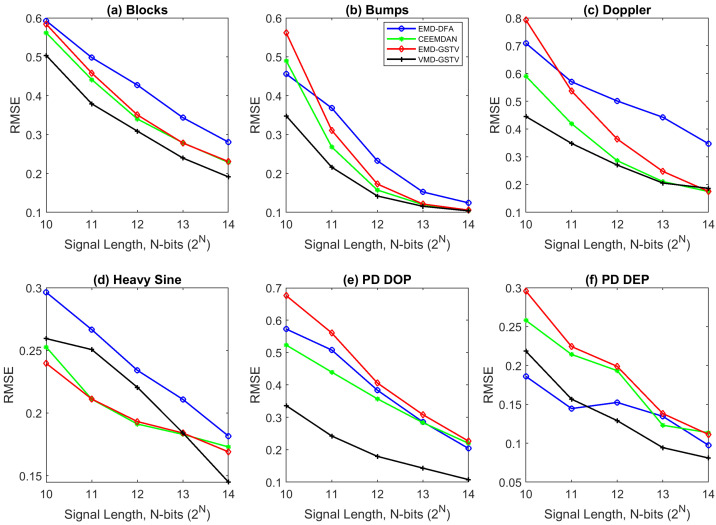
The RMSE value of various denoising algorithms at different signal length ($N=2^{10}$ to $2^{14}$) of synthetic test signals corrupted with 10 dB white noise.

Table 4, Table 5, Table 6 and Table 7 show the RMSE value computed between the denoised signal input signal, Figure 12 and Figure 13 show that in most of the cases the RMSE value of proposed VMD-GSTV is better than other methods. In certain cases the RMSE values of CEEMDAN is better than the proposed method, however, in the higher signal lengths the proposed method performs better. The proposed methods result in lower RMSE value for PD DOP signal, on the other hand PD DEP in lower noise levels with a higher signal length.

### 6.2. Illustration of the Denoising PD Signal

**Figure 14:**
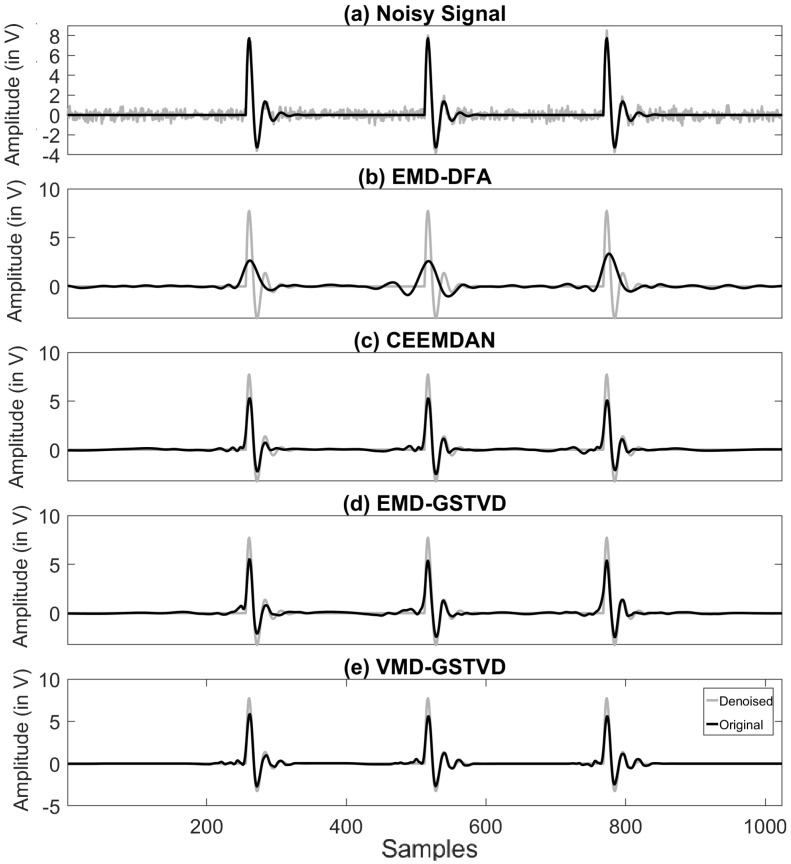
Input and reconstructed (**a**) synthetic ‘PD DOP’ signal and (**b**–**e**) reconstructed signal using various algorithms with signal length $N=2^{10}$.

**Figure 15:**
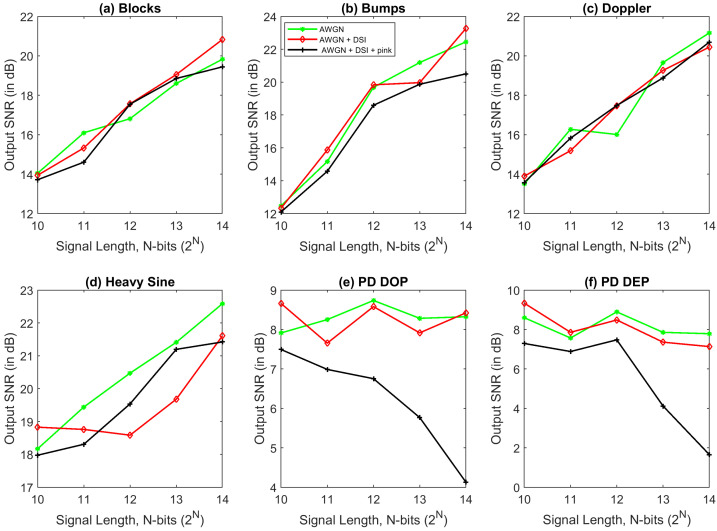
The output SNR value of proposed VMD-GSTV method for 5 dB AWGN, 5 dB AWGN+DSI, and 5 dB AWGN + DSI + pink noise at a different signal length ($N=2^{10}$ to $2^{14}$) of synthetic test signals.

The PD signals stated in Section 4.2 with different amplitude, damping factor, and damping frequencies were considered subject to denoising algorithms. The PD DOP signal corrupted with 10 dB AWGN and the denoised signal using various algorithms is shown in Figure 14. The most common source of noise described in Section 4.4, is mixed with a different PD signal, which adversely affects the shape of the original pulse shape and the PD footprints. Hence, the proposed algorithm is subjected to the noise models and the outcome is analyzed. Figure 15 shows the performance of VMD-GSTV on various test signals with a different signal length corrupted with AWGN of 5 dB or AWGN of 5 dB + DSI or AWGN of 5 dB + DSI + pink noise. It is observed that the four test signals have almost similar results, whereas the output SNR value of PD DOP and PD DEP signals have a down trend in output SNR while pink noise is added to the signal. However, as per the numerical values, the VMD-GSTV is better than other methods, which is not included in Figure 15.

**Figure 16:**
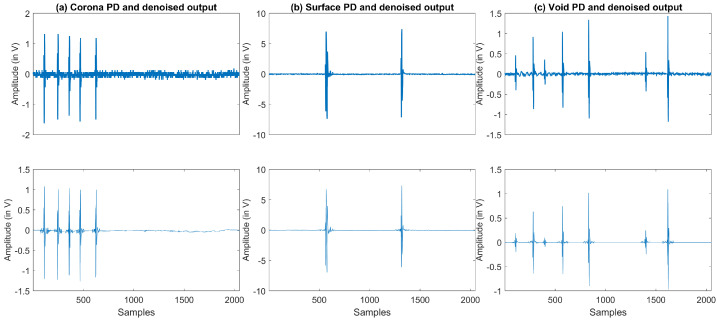
Denoised PD data using tje proposed VMD-GSTV method.

Apart from simulated PD signals, the real signals such as ‘PD Surface’, ‘PD Void’, and ‘PD Corona’ are also used in this work as a dataset from [44]. The data available in the literature is longer, hence only 2 K sample points are used in order to show the PD pulses on the plot. The output of the VMD-GSTV method is presented in Figure 16. The proposed method can eliminate the noise from the PD signal and retain the PD pulses.

## 7. Conclusions

In this paper, a new denoising method is proposed which combines VMD, statistical features such as MI, DE, and the GSTV denoising algorithm. Our approach is to decompose the signal using VMD with the mode parameter set by number of EMD IMFs with MI analysis, later VMD IMFs are also analyzed using MI to group noise-dominant and signal-dominant IMFs. Based on the DE values of the noise-dominant IMFs, the IMFs are filtered using GSTV with appropriate λ value to create the output vector. The signal-dominant IMFs are directly added to the output vector to create the denoised signal.

Using simulated and real data, the proposed method VMD-GSTV can remove the noise in the given signal while retaining the signal features. The proposed method is having better output SNR, RMSE and CC parameters in most signals as compared to the other methods considered in this paper. The output SNR of the proposed method is 4 to 13% higher than other methods for the input SNR of −5 dB, this indicates that the proposed method performs better under extreme noisy conditions. Furthermore, this method is applied to denoise synthetic PD DOP and PD DEP signal and real PD data of corona, surface, and void discharges. As per the observation, the proposed method has the ability to denoise the PD signals with low amplitude, buried white noise, DSI, and pink noise.

The application of other entropy methods such as Multiscale Dispersion Entropy (MDE), Refined Composite Multiscale Dispersion Entropy (RCMDE), and other decomposition methods such as ALIF and Empirical Wavelet Transform (EWT) will be considered in future work.

## Figures and Tables

**Table 1:** Mean frequency of each IMFs of Figure 4 decomposed using EMD and VMD method.

Method	${\mathrm{IMF}}_{1}$	${\mathrm{IMF}}_{2}$	${\mathrm{IMF}}_{3}$	${\mathrm{IMF}}_{4}$	${\mathrm{IMF}}_{5}$	${\mathrm{IMF}}_{6}$	${\mathrm{IMF}}_{7}$	${\mathrm{IMF}}_{8}$
EMD	351.50	145.04	41.37	25.19	11.66	8.07	5.01	3.20
VMD	0.92	8.26	33.94	64.31	242.86	338.66	396.83	459.97

**Table 2:** MI of phase spectra of consecutive IMFs of Figure 4 decomposed using EMD and VMD method.

Method	${\mathrm{IMF}}_{1-2}$	${\mathrm{IMF}}_{2-3}$	${\mathrm{IMF}}_{3-4}$	${\mathrm{IMF}}_{4-5}$	${\mathrm{IMF}}_{5-6}$	${\mathrm{IMF}}_{6-7}$	${\mathrm{IMF}}_{7-8}$	${\mathrm{IMF}}_{8-\mathrm{res}}$
EMD	0.0220	0.1029	0.6500	**2.3165**	4.3058	5.5138	8.0675	8.2695
VMD	**1.4764**	0.3018	0.3041	0.6974	0.6415	0.6618	0.3225	-

**Table 3:** Simulated Noise Signals.

Noise Type	S1	S2	S3	S4	S5	S6	S7	S8	S9	S10	S11
AWGN (S*a*) with SNR value	−5	0	5	10	20	5	10	20	5	10	20
DSI (S*d*) as per Equation (17).	-	-	-	-	-	Y	Y	Y	Y	Y	Y
Pink Noise (S*p*)	-	-	-	-	-	-	-	-	Y	Y	Y

**Table 4:** Filter evaluation parameters obtained from various denoising methods of 16 K samples ‘Block’ signal corrupted with AWGN. The best performance indicators are in bold font.

Input SNR	Parameter	Algorithms Applied to Synthetic Block Signal			
		EMD-DFA	CEEMDAN	EMD-GSTV	VMD-GSTV
−**5 dB**	SNR/dB	10.00	11.57	11.12	**13.07**
	RMSE	0.9471	0.7859	0.8299	**0.6754**
	CC	0.9510	0.9658	0.9620	**0.9735**
**0 dB**	SNR/dB	14.69	15.41	14.69	**15.96**
	RMSE	0.5502	0.5061	0.5496	**0.5046**
	CC	0.9827	**0.9854**	0.9829	0.9833
**5 dB**	SNR/dB	18.08	19.35	19.09	**19.83**
	RMSE	0.3709	0.3212	0.3304	**0.3094**
	CC	0.9922	0.9941	0.9938	**0.9943**
**10 dB**	SNR/dB	20.93	23.12	22.76	**24.35**
	RMSE	0.2667	0.2073	0.2161	**0.1804**
	CC	0.9960	0.9976	0.9973	**0.9981**
**20 dB**	SNR/dB	23.68	25.81	25.72	**28.53**
	RMSE	0.1965	0.1540	0.1540	**0.1121**
	CC	0.9978	0.9986	0.9986	**0.9993**

**Table 5:** Filter evaluation parameters obtained from various denoising methods of 16 K samples ‘Bumps’ signal corrupted with AWGN. The best performance indicators are in bold font.

Input SNR	Parameter	Algorithms Applied to Synthetic Bumps Signal			
		EMD-DFA	CEEMDAN	EMD-GSTV	VMD-GSTV
−**5 dB**	SNR/dB	10.60	11.32	11.72	**12.24**
	RMSE	0.5411	0.4935	**0.4696**	0.4759
	CC	0.9567	0.9638	**0.9674**	0.9628
**0 dB**	SNR/dB	15.81	16.91	15.88	**18.84**
	RMSE	0.2945	0.2608	0.2902	**0.2077**
	CC	0.9865	0.9893	0.9871	**0.9932**
**5 dB**	SNR/dB	20.16	21.16	21.13	**22.46**
	RMSE	0.1788	0.1588	0.1597	**0.1387**
	CC	0.9950	0.9961	0.9960	**0.9969**
**10 dB**	SNR/dB	24.54	26.51	26.05	**27.08**
	RMSE	0.1077	**0.0863**	0.0904	0.0870
	CC	0.9982	**0.9988**	0.9987	0.9986
**20 dB**	SNR/dB	32.96	33.73	34.21	**35.96**
	RMSE	0.0406	0.0371	0.0351	**0.0288**
	CC	0.9997	0.9998	0.9998	**0.9999**

**Table 6:** Filter evaluation parameters obtained from various denoising methods of 16 K samples ‘Doppler’ signal corrupted with AWGN. The best performance indicators are in bold font.

Input SNR	Parameter	Algorithms Applied to Synthetic Doppler Signal			
		EMD-DFA	CEEMDAN	EMD-GSTV	VMD-GSTV
−**5 dB**	SNR/dB	10.24	11.50	10.97	**12.89**
	RMSE	0.9127	0.7901	0.8306	**0.6796**
	CC	0.9502	0.9634	0.9630	**0.9747**
**0 dB**	SNR/dB	14.45	15.88	15.52	**16.35**
	RMSE	0.5581	0.4719	0.4930	**0.4610**
	CC	0.9822	**0.9873**	0.9861	0.9869
**5 dB**	SNR/dB	17.14	20.62	20.34	**21.17**
	RMSE	0.4113	0.2730	0.2826	**0.2582**
	CC	0.9900	0.9957	0.9954	**0.9961**
**10 dB**	SNR/dB	18.88	24.87	**25.05**	25.01
	RMSE	0.3486	0.1680	**0.1644**	0.1672
	CC	0.9922	0.9983	**0.9984**	0.9983
**20 dB**	SNR/dB	18.67	31.27	30.07	**32.64**
	RMSE	0.3712	0.0803	0.0946	**0.0686**
	CC	0.9908	0.9996	0.9994	**0.9997**

**Table 7:** Filter evaluation parameters obtained from various denoising methods of 16 K samples ‘Heavy Sine’ signal corrupted with AWGN. The best performance indicators are in bold font.

Input SNR	Parameter	Algorithms Applied to Synthetic Heavy Sine Signal			
		EMD-DFA	CEEMDAN	EMD-GSTV	VMD-GSTV
−**5 dB**	SNR/dB	10.80	11.78	11.33	**12.63**
	RMSE	0.9018	0.7975	0.8428	**0.7799**
	CC	0.9592	0.9682	0.9645	**0.9655**
**0 dB**	SNR/dB	16.25	15.57	15.31	**17.26**
	RMSE	0.4801	0.5155	0.5312	**0.4452**
	CC	0.9880	0.9864	0.9856	**0.9885**
**5 dB**	SNR/dB	20.03	20.25	20.27	**22.59**
	RMSE	0.3109	0.3013	0.3014	**0.2335**
	CC	0.9949	0.9953	0.9952	**0.9971**
**10 dB**	SNR/dB	24.99	26.15	25.59	**27.29**
	RMSE	0.1767	0.1522	0.1631	**0.1406**
	CC	0.9983	**0.9988**	0.9986	**0.9988**
**20 dB**	SNR/dB	32.78	32.35	34.83	**35.04**
	RMSE	0.0713	0.0746	0.0560	**0.0555**
	CC	0.9997	0.9997	**0.9998**	**0.9998**

## Data Availability

No new data were created in this study.

## Abbreviations

The following abbreviations are used in this manuscript:

ADMM	Alternating direction method of multipliers
ADTCWT	Adaptive dual tree complex wavelet transform
ALIF	Adaptive local iterative filtering
AM	Amplitude modulation
AWGN	Additive white Gaussian noise
CBWS	Correlation based wavelet selection
CC	Correlation coefficient
CEEMDAN	Complete ensemble empirical mode decomposition with adaptive noise
DE	Dispersion entropy
DEP	Damped exponential pulse
DOP	Damped oscillatory pulse
DSI	Discrete spectral interference
EBWS	Energy based wavelet selection
EEMD	Ensemble empirical mode decomposition
EMD	Empirical mode decomposition
EMD-DFA	EMD-Detrended fluctuation analysis
FM	Frequency modulation
GSTV	Group-sparse total variation denoising
HFCT	High frequency current transformer
HV	High voltage
IMF	Intrinsic mode function
MDE	Multiscale Dispersion Entropy
MI	Mutual information
NCDF	Normal cumulative distribution function
PD	Partial discharge
PE	Permutation Entropy
RCMDE	Refined Composite Multiscale Dispersion Entropy
RMSE	Root mean square error
TV	Total variation
TVD-MM	Majorization minimization based total variation denoising
VMD	Variational mode decomposition
WATV	Wavelet total variation
SNR	Signal-to-noise ratio
SNRBWS	SNR based wavelet selection
WPT	Wavelet packet transform
